# Maternal And preGnancy hEalth duriNg elevaTed heAt – MAGENTA: Novel data linkages and methods to understand the impacts of climate change on deprived communities in Wales and London.

**DOI:** 10.23889/ijpds.v9i5.2571

**Published:** 2024-09-18

**Authors:** Rich Fry, Rhiannon Owen, Samantha Turner, Amy Mizen, Lucy Griffiths, Pia Hardelid, Cathy Thornton

**Affiliations:** 1 Swansea University; 2 UCL

## Background and Objectives

The health impacts from changing climate, including increasing frequency, severity, and duration of heat are projected to worsen. The MAGENTA study will explore the impact of heat on pregnancy-related outcomes for deprived communities in Wales and London.

## Approach

MAGENTA will create linked data cohorts capturing some of the most deprived and diverse communities in Europe to analyse the impact deprivation, ethnicity, heatwaves, and urban heat islands have on pregnancy-related health. A consented data-linked cohort of pregnant women will provide biological samples and undergo direct measurement of themselves and their home environments to understand the heat stress response of pregnant women not acclimatised to heat during their pregnancy. Core to the study is our public involvement strategy which includes pregnant women and women who have recently delivered a baby, co-producing the research and how results are communicated.

## Results

Our electronic cohorts cover >10 years of climate and health data and capture all births in Wales >300,000 and >1.1 million in London.  We will present how the novel data linkages can work in multiple trusted research environments, approaches for sharing methods in geospatial modelling and statistics across the project and preliminary findings from the study.

## Findings and Implications

The MAGENTA study will help inform expectant parents, families and policy makers how to prepare for climate change in temperate regions. The comparison between different populations in Wales and London will give insight on where policies and interventions can be generally applied and where they need to be contextually adapted.

